# Acupuncture treatment for insulin sensitivity of women with polycystic ovary syndrome and insulin resistance: a study protocol for a randomized controlled trial

**DOI:** 10.1186/s13063-017-1854-2

**Published:** 2017-03-09

**Authors:** Juan Li, Ernest Hung Yu Ng, Elisabet Stener-Victorin, Zhenxing Hu, Xiaoguang Shao, Haiyan Wang, Meifang Li, Maohua Lai, Changcai Xie, Nianjun Su, Chuyi Yu, Jia Liu, Taixiang Wu, Hongxia Ma

**Affiliations:** 1 Department of Traditional Chinese Medicine, the First Affiliated Hospital of Guangzhou Medical University, Guangzhou, China; 2 Department of Obstetrics and Gynecology, the University of Hong Kong, Hong Kong Special Administrative Region, Hong Kong, China; 3 Department of Physiology and Pharmacology, Karolinska Institute, Stockholm, Sweden; 4 Department of Gynecology, Xuzhou Maternity & Child Health Hospital, Xuzhou, China; 5 Reproductive and Genetic Medical Center, Dalian Municipal Women and Children’s Medical Center, Dalian, China; 6 Department of Acupuncture and Moxibustion, Guangdong Provincial Hospital of Chinese Medicine, Guangzhou, China; 7 Department of Reproductive Health and Infertility, Guangdong Women and Children Hospital, Guangzhouᅟ, China; 8 Chinese Clinical Trial Registry, Beijing, China

**Keywords:** Acupuncture, Metformin, Sham acupuncture, Polycystic ovary syndrome, Insulin resistance

## Abstract

**Background:**

Our prospective pilot study of acupuncture affecting insulin sensitivity on polycystic ovary syndrome (PCOS) combined with insulin resistance (IR) showed that acupuncture had a significant effect on improving the insulin sensitivity of PCOS. But there is still no randomized controlled trial to determine the effect of acupuncture on the insulin sensitivity in women with PCOS and IR. In this article, we present the protocol of a randomized controlled trial to compare the effect of true acupuncture on the insulin sensitivity of these patients compared with metformin and sham acupuncture. Acupuncture may be an effective therapeutic alternative that is superior to metformin and sham acupuncture in improving the insulin sensitivity of PCOS combined with IR.

**Methods:**

This study is a multi-center, controlled, double-blind, and randomized clinical trial aiming to evaluate the effect of acupuncture on the insulin sensitivity in PCOS combined with IR. In total 342 patients diagnosed with PCOS and IR will be enrolled. Participants will be randomized to one of the three groups: (1) true acupuncture + metformin placebo; (2) sham acupuncture + metformin, and (3) sham acupuncture + metformin placebo. Participants and assessors will be blinded. The acupuncture intervention will be given 3 days per week for a total of 48 treatment sessions during 4 months. Metformin (0.5 g per pill) or placebo will be given, three times per day, and for 4 months.

Primary outcome measures are changes in homeostasis model assessment of insulin resistance (HOMA-IR) and improvement rate of HOMA-IR by oral glucose tolerance test (OGTT) and insulin releasing test (Ins). Secondary outcome measures are homeostasis model assessment-β (HOMA-β), area under the curve for glucose and insulin, frequency of regular menstrual cycles and ovulation, body composition, metabolic profile, hormonal profile, questionnaires, side effect profile, and expectation and credibility of treatment.

Outcome measures are collected at baseline, at the end of treatments, and 3 months after the last acupuncture treatment. On completion of the screening visit, randomization will be conducted using a central randomization system.

**Discussion:**

This study will investigate the effects of acupuncture on the insulin sensitivity of PCOS and IR women compared with metformin and sham acupuncture. We will test whether true acupuncture with needles placed in skeletal muscles and stimulated manually and by electrical stimulation is more effective than metformin and sham acupuncture with superficial needle placement with no manual or electrical stimulation in improving the insulin sensitivity in PCOS women with IR.

**Trial registration:**

ClinicalTrials.gov, NCT02491333; Chinese Clinical Trial Registry, ChiCTR-ICR-15006639. Registered on 24 June 2015.

**Electronic supplementary material:**

The online version of this article (doi:10.1186/s13063-017-1854-2) contains supplementary material, which is available to authorized users.

## Background

Polycystic ovary syndrome (PCOS) is one of the most common endocrine disorders in reproductive age women. It is characterized by chronic anovulation and hyperandrogenism and is often accompanied by obesity and insulin resistance (IR) as IR is present in nearly 40% of PCOS [1]. In addition, IR, hyperinsulinemia, and dyslipidemia worsen with aging, and the risk of miscarriage is three times higher than in healthy women [2]. They are also at an increased risk of pregnancy complications such as impaired glucose tolerance, gestational diabetes mellitus, pregnancy-induced hypertension and preeclampsia, and small for gestational age children [2]. It may affect general health and the quality of life. Both hyperandrogenism and IR contribute to the pathogenesis of many aspects of PCOS [3, 4].

IR in PCOS results from hyperinsulinemia and defects in the insulin signaling pathway. Further, high concentrations of insulin reduce circulating levels of sex hormone binding globulin (SHBG) and increase levels of free testosterone, and the latter leads to menstrual disturbance, development of ovarian cysts, hirsutism, and anovulatory infertility [5]. IR leads to a higher risk of impaired glucose, type 2 diabetes, dyslipidemia, atherosclerosis, and vascular disease [6].

The first-line treatment for overweight or obese women with PCOS is modification of diet and lifestyle. This confirmed that the benefit of improved ovulation and live birth with delayed infertility treatment with clomiphene citrate when preceded by lifestyle modification with weight loss was superior to immediate treatment [7], but for many subjects it is not easy to sustain [8]. Treatments of IR with insulin-sensitizing agents have been used in women with PCOS [9]. Metformin, an insulin-sensitizing agent known to decrease hepatic glucose production via a transient inhibition of the mitochondrial respiratory-chain complex 1 and an activation of the AMP-activated protein kinase (a cellular metabolic sensor [10]), improves irregular menstrual cycles in women with PCOS whether IR is present or not [11]. Nevertheless, it is not effective in treating hirsutism, acne, or anovulatory infertility [12]. It also has side effects such as gastrointestinal irritation (diarrhea and nausea) and lactic acidosis, which is rare but serious [13]. In addition, metformin is not recommended for patients with kidney, lung, liver, and heart disease or on a low-carbohydrate diet [14]. Other common insulin-sensitizing agents are thiazolidinediones, including troglitazone (which is no longer available for clinical use), rosiglitazone, and pioglitazone [15]. However, rosiglitazone may result in cardiovascular diseases [16], and pioglitazone may increase the risk of bladder cancer [17]. Both may double the risk of bone fractures [18]. Given that current pharmacological treatments have such side effects, there is an urgent need to find a better and safer therapy for IR in PCOS women.

As an integrated part of Traditional Chinese Medicine and a relatively safe treatment [19], acupuncture treatment for PCOS has gained increasing attention in recent years. The effect of acupuncture is most likely mediated via activation of sensory nerve fibers, which in turn modulate the sympathetic activity to the ovaries and in the central nervous system [20, 21]. A review found that acupuncture can correct various metabolic disorders contributing to the development of IR, such as hyperglycemia, overweight, hyperphagia, hyperlipidemia, inflammation, altered activity of the sympathetic nervous system, and insulin signal defect [22]. Thus, acupuncture may have the potential to improve insulin sensitivity [22]. Other findings suggest that electrical acupuncture stimulation affects more functional signaling pathways related to insulin sensitivity, while manual stimulation of acupuncture needles has a greater effect on glucose tolerance [23]. Furthermore, in female insulin-resistant rats both electrical and manual acupuncture stimulation enhances insulin sensitivity via activation of sensory afferents [24]. Our prospective pilot study (ClinicalTrials.gov NCT02026323) [25] of acupuncture affecting the insulin sensitivity in 81 cases of women with PCOS with IR showed that acupuncture has a significant effect on improving the insulin sensitivity in these patients. But there is still no randomized controlled trial to determine the effect of acupuncture on insulin sensitivity in women with PCOS combined with IR.

Thus, we present the protocol of a randomized controlled trial to investigate the effects of acupuncture on the insulin sensitivity of women with PCOS combined with IR. We hypothesize that true acupuncture is superior to metformin and sham acupuncture in improving insulin sensitivity.

### Objectives

The primary objective of this randomized controlled trial is to evaluate the hypothesis that true acupuncture + metformin placebo (group 1) improves insulin sensitivity, as measured by changes in the homeostasis model assessment of insulin resistance (HOMA-IR), more effectively than sham acupuncture + metformin (group 2) and sham acupuncture + metformin placebo (group 3) in women with PCOS and IR. Secondary objectives include changes in HOMA-β (islet β-cell function), C-peptide index, the insulin response to glucose calculated from the oral glucose tolerance test (OGTT) and insulin releasing test (Ins), frequency of the regular menstrual cycle and ovulation, circulating sex steroids, lipid profile, health-related quality of life and symptoms of anxiety and depression, side effects, and credibility.

## Methods

### Overview

This is a randomized controlled trial to determine the effect of true acupuncture + metformin placebo for the improvement of insulin sensitivity in women with PCOS and IR compared with sham acupuncture + metformin and sham acupuncture + metformin placebo. The design of this study is compliant with the Consolidated Standards of Reporting Trials (CONSORT) guidelines [26] and with the Standard Protocol Items: Recommendations for Interventional Trials (SPIRIT) statement 2013 [27] [see Additional file 1].

### Patients and enrollment

**Fig. 1:**
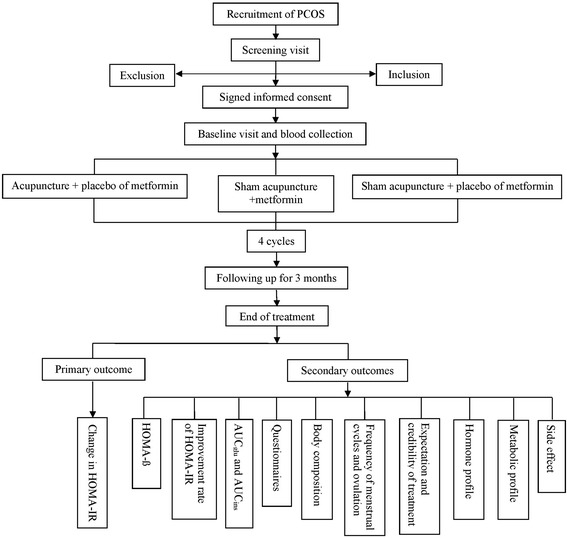
CONSORT flow diagram of the study. *PCOS* polycystic ovary syndrome; *HOMA* homeostatic model assessment; *IR* insulin resistance; *AUC* area under the curve

Women with PCOS and IR will be recruited from the Department of Traditional Chinese Medicine in the First Affiliated Hospital of Guangzhou Medical University, Xuzhou Maternity & Child Health Hospital, Dalian Municipal Women and Children’s Medical Center, Guangdong Women and Children’s Hospital, *and* Hexian Memorial Affiliated Hospital of Southern Medical University if they meet the inclusion criteria and do not have any exclusion criteria. Eligible subjects will be approached and sign the consent form after detailed explanation of the study design and comprehensive counseling (see the flow diagram in Fig. 1).

#### Inclusion Criteria

1. Chinese women aged from 18 to 40 years.
2. ﻿Body mass index ﻿(BMI) ≥18.5 kg/m^2^.
3. Confirmed diagnosis of PCOS according to the Rotterdam criteria in 2003 including at least two of the following three features: (1) oligo- (an intermenstrual interval >35 days or <8 cycles in the past year) or amenorrhea (an intermenstrual interval >90 days), and/or (2) polycystic ovarian morphology, i.e., presence of >12 antral follicles (≤9 mm) and/or ovarian volume >10 ml on ultrasonography scanning, and/or (3) clinical and/or biochemical hyperandrogenism. Clinical hyperandrogenism on the Chinese Mainland is defined as a Ferriman-Gallwey (FG) score ≥5 [28]; biochemical hyperandrogenism is total testosterone (T) >2.6 nmol/l and free testosterone ≥6.0 pg/ml [29].
4. Presence of IR as defined by the homeostatic model assessment—HOMA-IR: [fasting insulin (μU/ml) × fasting glucose (mmol/l)] / 22.5). A value ≥2.14 is considered to be indicative of IR [30].
5. No immediate fertility wish and willingness to use barrier contraceptive methods for 7 months.
6. Willingness to sign the consent form.

#### Exclusion Criteria

1. Exclusion of other endocrine disorders:
  1. Uncorrected thyroid disease (defined as thyroid stimulating hormone (TSH) < 0.2 mIU/ml or >5.5 mIU/ml, triiodothyronine (T3) < 1.4 nmol/l or >2.2 nmol/l, and free thyroxine (T4)  < 10 pmol/l or >23 pmol/l). A normal level within the last year is adequate for entry.
  2. Poorly controlled type I or type II diabetes (defined as a glycosylated hemoglobin (HbA1c) level >7.0%) or patients receiving antidiabetic medications such as insulin, thiazolidinediones, acarbose, or sulfonylureas likely to confound the effects of the study medication; patients currently receiving metformin XR (extended release) for a diagnosis of type I or type II diabetes or for PCOS are also excluded.
  3. Cushing’s syndrome (defined as an archetype of metabolism syndrome. High glucocorticoid levels lead to muscle, liver, and adipocyte insulin resistance; 17-hydorxycorticosteroids >55 umol/24 h or urinary-free cortisol >304 nmol/24 h).
  4. Congenital adrenal hyperplasia (defined as patients with known 21-hydroxylasedeficiency or other enzyme deficiency leading to the phenotype of congenital adrenal hyperplasia; 17-oh progesterone >10 ng/ml in adrenocorticotropic hormone 1-24 h excited test (after 60 min).
  5. Suspected androgen-secreting adrenal or ovarian tumor.
2. Use of hormonal or other medication including Chinese herbal prescriptions, which may affect the outcome of the last 2 months.
3. Receiving acupuncture in the past 2 months.
4. Within 6 weeks of pregnancy.
5. Post-abortion or postpartum within the past 6 weeks.
6. Breastfeeding within the last 4 months.
7. Not willing to give written consent to the study.
8. Having a bariatric surgery procedure within the past 12 months or being in a period of acute weight loss.
9. Additional exclusion criteria include:
  - Patients on oral contraceptives, depot progestin, or hormonal implants (including Implanon). A 2-month washout period will be required prior to screening for patients on these agents. Longer washouts may be necessary for certain depot contraceptive forms or implants, especially where the implants are still in place. A 1-month washout will be required for patients on oral cyclic progestin.
  - Heart disease.
  - Patients with a history of or suspected cervical carcinoma, endometrial carcinoma, or breast carcinoma.
  - Patients enrolled in other studies that require medications.
  - Patients taking longer than a 1-month break during the protocol should not be enrolled.

### Intervention arms

#### Information to all study subjects

All participants will be advised about the importance of regular physical exercise and a balanced diet by a trained dietician before they receive the treatments. Importantly, they are instructed not to change their exercise or diet habits during the entire study period.

True acupuncture or sham acupuncture and metformin will be started 2 days after the baseline visit including OGTT and Ins. All subjects will be asked to use a barrier method for contraception. True and sham acupuncture treatment will be given three times per week. Each treatment session lasts for 30 min and can be separated by an interval of 1-3 days, with a maximum of 48 treatment sessions during 4 months. After the third treatment, all patients fill in the expectation and credibility questionnaire [31]. Metformin (0.5 g per pill) or placebo will be given, three times per day for 4 months.

### True acupuncture protocol

The rationale of acupuncture protocols is based on traditional Chinese and Western medical theories, and the study protocol follows the CONSORT [26] and Standards for Reporting Interventions in Clinical Trials of Acupuncture (STRICTA) [32] recommendations. We will use fixed acupuncture protocols. The acupuncture protocol of this randomized controlled trial (RCT) follows the protocol in the previous RCT in women with PCOS including *Acupuncture to Treat Insulin Resistance in Women With and Without Polycystic Ovary Syndrome* (ClinicalTrials.gov NCT01457209) [33], *Acupuncture and Clomiphene Citrate on Live Birth in Anovulatory Women With Polycystic Ovary Syndrome* (ClinicalTrials.govNCT01573858) [34], and a prospective pilot study of *the Effect of Acupuncture on Insulin Sensitivity Polycystic Ovary Syndrome* (ClinicalTrials.gov NCT02026323) [25].

Disposable, single-use, sterilized needles made of stainless steel, 0.25 × 30 mm and 0.30 × 40/50 mm (Huanqiu, Suzhou Huanqiu Acupuncture Medical Instrument Co., Ltd. 215002 Suzhou, China), will be inserted to a depth of 15–35 mm in segmental acupuncture points located in abdominal and leg muscles with innervations corresponding to the ovaries. Two sets of acupuncture points will be alternated every second treatment (Table 1 [25]). The first set consists of the conception vessel (CV) 3, CV 12, and stomach (ST) 29 bilaterally and in the muscles above the knee, ST34, and ST 33 bilaterally and below the knee, spleen (SP) 6, and ST 36. Needles will also be placed in extra-segmental acupuncture points that do not innervate the ovaries large intestine (LI) 4 bilaterally. In total, 14 needles will be placed, and all will be stimulated manually by rotating the needle to evoke needle sensation (de *qi*) once when inserted. The following points will be connected to an electrical stimulator (Export Abteilung, Schwa-Medico GmbH, Wetzlarer Str. 41-43; 35630 Ehringshausen): CV 3 to CV 12, ST 29 bilateral, and ST 34 to ST 33 bilateral. Stimulations are given as low-frequency electric acupuncture (EA) of 2Hz, 0.3 ms pulse length, with an intensity adjusted to produce local muscle contractions without pain or discomfort. Needles not connected to the electrical stimulator will be manually stimulated to evoke needle sensation every 10 min, in total four times. The second set consists of 14 needles placed in segmental abdominal points and stimulated electrically: ST 27 bilaterally, CV 6 connected to CV 10; leg points: SP10 connected to a non-acupuncture point located 6 cun proximal to the patella as the medial border (electrical stimulation); SP 6 and liver (LR) 3 bilaterally (manual stimulation). Extra-segmental points are pericardium (PC) 6 bilaterally (manual stimulation).

**Table 1:** Acupuncture protocol [25]

Point	Stimulation	location	Muscle	Innervation
Set 1				
Zhongji (CV3)	EA	4 cun caudal to the umbilicus	Fibrous tissue, linea alba	L1
Zhongwan (CV12)	EA	On the midline, 4 cun superior to the umbilicus	Fibrous tissue, linea alba	Th7-8
Guilai (bilateral) (ST29)	EA	1 cun cranial to the pubic bone and 2 cun lateral of the midline	M. rectus abdominis	Th6-12
Liangqiu (bilateral) (ST34)	EA	2 cun above the superior lateral border of the patella on the line connecting the anterior superior iliac spine found with the knee flexed	M. quadriceps femoris	Femoral nerve
Yinshi (bilateral) (ST33)	EA	3 cun above the superior lateral border of the patella on the line connecting the anterior superior iliac spine found with the knee flexed	M. quadriceps femoris	Femoral nerve
Sanyinjiao (bilateral) (SP6)	De *Qi* four times	3 cun proximal to the medial malleolus	Mm. flexor digitorum longus, tibialis posterior	L4–5, S1–2
Zusanli (bilateral) (ST36)	De *Qi* four times	On the anterior lateral side of the leg, 3 cun below Dubi (ST35), one finger width (middle finger) from the anterior crest of the tibia	Musculi tibialis anterior	L4–5, S1
Hegu (bilateral) (LI4)	De *Qi* four times	On the highest point at m. interosseus dorsalis	Mm. interosseus dorsalis I, lumbricalis II, adductor pollicis	C8, Th1
Set 2				
Daju (bilateral) (ST27)	EA	3 cun cranial to the pubic bone and 2 cun lateral to the midline	M. rectus abdominis	Th6–12
* Qi*hai (CV6)	EA	1.5 cun caudal to the umbilicus	Fibrous tissue, linea alba	Th11
Xiawan (CV10)	EA	2 cun cranial to the umbilicus	Fibrous tissue, linea alba	Th8
Extra-meridian point (bilateral)	EA	6 cun above the patella in line with SP10	M. quadriceps femoris	L2–L4
Xuehai (bilateral) (SP10)	EA	With the knee flexed, on the medial side of the thigh 2 cun above the superior medial corner of the patella on the prominence of the medial head of the quadriceps muscle of the thigh	M. quadriceps femoris	L2–L4
Sanyinjiao (bilateral) (SP6)	De *Qi* four times	3 cun proximal to the medial malleolus	Mm. flexor digitorum longus, tibialis posterior	L4–5, S1–2
Taichong (bilateral) (LR3)	De *Qi* four times	Between metatarsal I & II, just distal to the caput	M. interosseus dorsalis I	S2–3
Neiguan (bilateral) (PC6)	De *Qi* four times	2 cun proximal to the processus styloideus radii, between the tendons of the palmaris longus and the flexor carpi radialis	M. flexor digitorum superficialis	C8, Th1

The two sets will be alternated for every other treatment

*C* cervical vertebra, *CV* conception vessel, *EA* electroacupuncture, *L* lumbar vertebra, *LI* large intestine, *LR* liver, *M.* musculi, *Mm.* musculus, *PC* pericardium, *S* sacral vertebra, *SP* spleen, *ST* stomach, *Th* thoracic vertebra

#### Needle insertion technique

Needle insertion should be gentle. The skin is tightened by pressing around the area of needle insertion, and the needle is then gently inserted.

#### Needles and stimulation

Needle size: 0.25 × 30 mm or 0.30 × 40 or 0.30 × 50 mm. Select needle length with respect to the patients’ BMI: 0.25 × 30-mm needles in women with normal BMI, 0.30 × 40-mm needles in overweight women, and 0.30 × 50 or 75-mm needles in obese women. Depth of insertion may vary from patient to patient. Needles are placed with a depth deep enough to reach muscle/fibrous tissue. When a needle is inserted, the needle is stimulated gently until de *qi* (needle sensation reflecting activation of sensory afferents). As soon as de *qi* has been reached, the needle does not hurt or cause any pain and discomfort. Manual stimulation of needles that are not attached to an electrical stimulator is stimulated when inserted, after 10 min, 20 min, and immediately before they are removed after 30 min.

The electrodes are attached to an electric stimulation according to the protocol. The electric stimulator is turned on to program 10, and the intensity is gradually increased. The intensity will be as high as possible without causing pain or discomfort of subjects. The stimulation amplitude/intensity will be adjusted after 10 and 20 min at the same time as when manually stimulated needles are manipulated. After 30 min the stimulator is turned off, electrodes are disconnected, and needles are taken out.

### Sham acupuncture protocol

Disposable, single-use, sterilized needles made of stainless steel, 0.20 × 20 mm (Huanqiu, Suzhou Huanqiu Acupuncture Medical Instrument Co., Ltd. 215002 Suzhou, China) will be inserted superficially to a depth of <5 mm, one in each shoulder and one in each upper arm at non-acupuncture points (Table 2 [34]) with no stimulation. Placement of needles is unlikely to affect ovulation and IR in women with PCOS. Electrodes will be attached to the needles, and the stimulator will be turned on at an intensity of zero (no active current) to mimic EA in the acupuncture protocol. No manual stimulation of the needles will be performed. An intended adjustment of the intensity is made after 10 min, 20 min, and again after 30 min when the stimulator is turned off and the needles removed.

**Table 2:** Sham acupuncture protocol [34]

Point	Stimulation	location	Skin innervation
No known point	Sham EA	On top of acromion	C3-4, n. supraclavicularis
No known point	Sham EA	On humerus, behind LI14	C5-6, n. cutaneous brachii lateralis

### True and sham acupuncture treatment

1. Time of the day and the name of the acupuncturist when the patient receives acupuncture will be recorded.
2. The intensity of stimulation (mA) is noted. It may vary between different electrodes. Note the range, e.g., 1.2 – 3.0 mA.
3. Note any other events that may affect the treatment (positive or negative).
4. Note concomitant medications.
5. Collect menstrual logs at the end of every cycle.

### Metformin and placebo

Metformin (Bristol-Myers Squibb Co., Shanghai, China) and placebo tablets (Jaden Pharmaceutical Co., Ltd., Guangzhou, China) will be packed and tested by a commercial pharmacy supply company (Panlongyunhai Pharmaceutical Co., Kunming, Yunnan, China) specifically for this study. Metformin or placebo will be started 2 days after the baseline visit including OGTT and Ins. Subjects will take 0.5 g/time, three times per day for 4 months. If the patients have adverse effects such as nausea and dizziness, the dose will be reduced to 0.5 g/time, two times or one time per day according to the severity.

### Monitoring and examination during the treatment

All subjects will be instructed not to change their physical exercise habits or diet during the entire study. They are asked to use barrier contraception, not hormonal contraception. Physical examination will be performed monthly. Every menstruation should be recorded, including the date, volume, and duration of menstruation during the entire study and follow-up. The conditions of physical exercise and diet will be recorded for every cycle.

### Study-specific visits and procedures

Patients will attend five visits, including the screening visit, baseline measurements, treatment visit, after-treatment measurements, and follow-up measurements. Months indicate when each specific measurement takes place. Adverse events and concomitant medications will be recorded during every visit. The overview of study visits is shown in Table 3.

**Table 3:** Overview of study visits

	Screening visit	Baseline visit	Month				Follow-up after 4 months of treatment	End of treatment visit 3 months after the last acupuncture treatment
			1st	2nd	3rd	4th		
OGTT and Ins		√					√	√
Body composition (weight, height, waist circumference, hip circumference, FG/acne)	√	√	√	√	√	√	√	√
Menstrual cycle diary	√	√	√	√	√	√	√	√
Fasting blood samples for FGLU, FINS, HbA1c, C-peptide, TSH, T3, free T4	√							
Fasting blood samples for FSH, LH, SHBG, T, E2, PRL		√					√	√
Fasting blood samples for ApoA1,ApoB, TC, TG, HDL-C, LDL-C, BRT, renal and liver profile		√					√	√
Transvaginal or transrectal ultrasound	√						√	√
Questionnaires		√					√	√

*Apoa1* apolipoprotein A1, *ApoB* apolipoprotein B, *BRT* blood routine test, *E2* estradiol, *FG* Ferriman-Gallwey score, ﻿*FGLU* f﻿asting blood glucose*, FINS* Fasting insulin, *FSH* follicle-stimulating hormone, *HbA1c* glycosylated hemoglobin, *HDL-C* high density lipoprotein, *LDL-C* low density lipoprotein, *LH* luteinizing hormone, *PRL* prolactin, *SHBG* sex hormone-binding globulin, *T* total testosterone, *TC* total cholesterol, *TG* triglyceride, *TSH* thyroid stimulating hormone, *T3* triiodothyronine, *T4 *thyroxine, *OGTT* oral glucose tolerance test, *Ins* insulin-releasing test

### Screening visit

Women are screened in the morning after an overnight 12-h fast. Detailed information about the study design is given.

#### Obtain signed informed consent

##### Complete physical examination

Complete physical examinations will be performed including height, weight, hip, and waist measurements. Height and weight will be recorded to the nearest 0.1 cun and 0.1 kg, respectively. Waist and hip circumference will be recorded to the nearest 1 cun. Hirsute assessment by FG, acne standard acne lesion counts, and pelvic examination will be conducted.

#### Perform transvaginal or transrectal ultrasound of ovaries

Ovaries, including the ovarian size in three dimensions, the size of the largest ovarian follicle/cyst and size of every follicle with a mean diameter greater than 10 mm, and total antral follicle count (small follicles with mean diameter <10 mm) of each ovary will be obtained through transvaginal or transrectal ultrasound.

#### Check urine pregnancy test

##### Fasting blood samples to exclude other endocrine disorders

Fasting (at least 4 h) blood samples are taken to exclude any endocrine disorders:

Fasting glucose, fasting insulin, HbA1C (>7%), C-peptide, TSH (<0.2 mIU/ml or >5.5 mIU/ml), T3 (<1.4 nmol/l or >2.2 nmol/l), and free T4 (<10 pmol/l or >23 pmol/l).

#### Progestin withdrawal

Provide progestin prescription to induce withdrawal bleeding, with instructions to begin medication once eligibility has been determined.

### Baseline visit

After the screening visit, if a woman fulfills inclusion criteria and has signed the informed consent form, she will be assigned to the baseline visit. The baseline visit takes place day 2–5 of a spontaneous period or after a withdrawal bleeding after an overnight fast.

#### Laboratory Examination

Blood will be collected. Part of it will be stored, and the remainder is used for determination of DNA and sphingolipid levels.

1. *Circulating sex steroids:* Fasting serum levels of follicle-stimulating hormone (FSH), luteinizing hormone (LH), estradiol (E2), prolactin (PRL) and T, and sex hormone binding globulin (SHBG).
2. *Lipid profile:* Triglycerides (TG), total cholesterol (TC), high-density lipoprotein (HDL-C), low-density lipoprotein (LDL-C), apolipoprotein A1 (ApoA1), apolipoprotein B (ApoB), blood routine test﻿(BRT), and renal and liver profiles.
3. *Glucose homeostasis and insulin sensitivity:* The OGTT and Ins with 75 g glucose. Blood samples will be obtained to measure plasma glucose, serum insulin, and C-peptide at 0, 60, and 120 min during the OGTT and Ins.

#### Complete questionnaires

Quality of life will be assessed by the short form 36 (SF-36) [35], the Chinese Quality of Life (ChiQOL) [36], sleeping questionnaires [37], international physical activity questionnaire (IPAQ) [38], questionnaire of eating and weight patterns (QEWP) [39], and the Polycystic Ovary Syndrome Questionnaire (PCOS-QOL) [40]. We will also assess symptoms of anxiety and depression by the Zung SAS and Zung SDS questionnaires and complete the quantization table of traditional Chinese medicine (TCM) syndromes about PCOS.

#### Assess TCM syndromes of patients

Syndrome differentiation (Bian Zheng) in TCM is the comprehensive analysis of clinical information gained by the four main diagnostic TCM procedures: observation, listening, questioning, and pulse analysis, and it is used to guide the choice of treatment by acupuncture and/or TCM herbal formulae [41]. In PCOS, patients are empirically differentiated into four categories: (1) phlegm-dampness syndrome, (2) blood stasis syndrome, (3) phlegm, and (4) blood stasis.

### Visit after 4 months of acupuncture treatment

During the end of treatment visit, all baseline measures will be repeated as listed below:

1. Physical examination will be performed, including vital signs, height, weight, and hip and waist measurements as well as repeating hirsute and acne assessments after the end of treatment or pregnancy.
2. The serum levels of sex hormone steroids and the metabolic profile will be repeated.
3. The transvaginal or transrectal ultrasound will be repeated for antral follicle counts.
4. OGTT and Ins will be repeated.
5. Blood will be collected for storage and for determination of DNA and sphingolipid levels.
6. QOL, sleeping, IPAQ, anxiety/depression questionnaires, QEWP, and the quantization table of TCM will be repeated.
7. Menstrual logs will be collected.
8. Adverse events and concomitant medications will be recorded.
9. The questionnaires about acupuncture treatment will be answered.

### End of treatment visit

The end of treatment visit will be done 3 months after the last acupuncture treatment (see points 1-8 under Visit after 4 months of acupuncture treatment). All the subjects will be followed up by visiting the acupuncturists monthly for 3 months. The conditions of physical exercise and diet will be record every month.

### Safety analysis

Adverse events will be categorized and the percentage of patients experiencing adverse events and serious adverse events during the treatment period and follow-up period will be documented and reported to the Data and Safety Monitoring Board (DSMB). These will be reviewed on a quarterly basis by the DSMB, and serious adverse events will be immediately reviewed.

### Outcome measures

#### Primary outcome

Change in HOMA-IR.

#### Secondary outcomes

1. Improvement rate of HOMA-IR.
2. HOMA-β: Islet β-cell function will be evaluated by the formula (20 × fasting insulin (mU/ml) / (fasting plasma glucose (mmol/l) − 3.5)) [42] and by the C-peptide index (CPI) Fasting C-peptide (nmol/l)/f-glucose (mmol/l) × 100 [43].
3. The insulin response to glucose will be assessed by calculating the area under the curve during the OGTT and Ins performance for glucose (AUCglu) and insulin (AUCins) using the trapezoidal rule [44].
4. Frequency of menstrual cycles and ovulation.
5. Body composition: weight, BMI, waist-to-hip circumference, and FG and acne lesion counts.
6. Metabolic profile: glucose and insulin concentrations, C-peptide, HbA1c, TC, TG, HDL-C and LDL-C, and ApoA1 and ApoB.
7. Hormonal profile: FSH, LH, T, and SHBG.
8. Questionnaires: SF-36, ChiQOL, sleeping questionnaires, IPAQ,QEWP, PCOS-QOL, Zung SAS, Zung SDS questionnaires, and the quantization table of TCM syndromes about PCOS will be used to assess the quality of life and sleep and the emotional state and to measure the moderate physical activity.
9. Side effect profile.
10. Expectation and credibility of treatment.

### Data management and quality control of data

Both the Case Record Form (CRF) and web-based electronic database will be used to manage individual participant data. Quality control of the data will be handled at two different levels: the investigators will be required to ensure the accuracy of the data as the first level of control when they input the records in CRF. The second level will include data monitoring and validation that will be carried out by an independent group. A web-based electronic database, ResMan (www.medresman.org), is used as a double input database. Finally, the individual participant data except the private information will be allowed to be share with the public within 6 months after the trial completion.

### Sample size calculations

In our pilot study [25] evaluating the effect of acupuncture on insulin sensitivity in PCOS women with IR, HOMA-IR reduced significantly after 3 months of acupuncture treatment when an interim analysis was performed and changed from (4.3 ± 2.5) vs. (3.7 ± 2.1). HOMA-IR ≥2.14 was considered to be abnormal. We anticipate that the HOMA-IR after 4 months of true acupuncture treatment will decrease 25%, and sham acupuncture will be decreased 5% as it is well known that sham is not an inert procedure. Therefore, the difference is 20%, with two-sided α assigned to be 5% and β 20% at the upper limit, respectively, at which the power is 80% and the rate of attrition estimated to be 20%. Thus, the sample size has been inflated from 95 to 114 per group, totaling 342 cases for the three arms. The sample size was calculated using the software statistics toolkit supported by the Department of Obstetrics and Gynecology of the Chinese University of Hong Kong (http://www.obg.cuhk.edu.hk/ResearchSupport/StatTools/index.php).

### Randomization and patient allocation

Subjects will be randomly allocated into one of the three treatment arms by a 1:1:1 treatment ratio: (1) true acupuncture + metformin placebo, (2) sham acupuncture + metformin, and (3) sham acupuncture + metformin placebo. The Data Coordination Centre (DCC) statisticians will generate the randomization scheme for the study. The true and sham acupuncture treatments will be known only to the acupuncturists besides the DCC data manager. The metformin and placebo having the same package will be organized in a kit consisting of 4 sacks for each subject with 336 tablets in total, respectively. The kit will be labeled with an ID number mapped to the metformin and placebo known only to the DCC personnel. The metformin and placebo assignments will be double-blind to any site investigators.

A central randomization system will be used to allocate patients. Patients will be stratified based on three sites and randomly assigned. The *Chinese Clinical Trial Registry* takes the responsibility for generation of the random number sequence by computer software, and the allocation sequence is deposited in ResMan, a web-based database. When a new participant enrolls, the allocation information will be obtained by inputting a special password in ResMan. It is not possible to speculate the allocation status of the participant before getting the information.

### Statistical analysis strategy

The Kolmogorov-Smirnov test will be used to test the normal distribution of continuous variables. Continuous variables will be presented by mean ± standard deviation if they are normally distributed or by median with interquartile range if they are not normally distributed. Both per protocol (PP) analysis and intention-to-treat analysis (ITT) will be used to determine the robustness of the evidence. Analysis of variance (ANOVA) with Bonferroni corrections will be used for comparing the differences between groups of continuous data in accordance with a normal distribution or Kruskal-Wallis test for non-normal distribution data when appropriate; χ^2^ tests will be used for comparison of dichotomous data. For the primary outcome, analysis of covariance (ANCOVA) (accompanying LSMEAN and 95% CI) will be used. ANOVA will be used to calculate the changes from baseline to after treatment and follow-up, respectively, and it will also be used to compare the differences between treatment arms with respect to the primary outcome, HOMA-IR. Effect of continuous data will be reported by both *P*-value and mean difference with 95% confidence interval and categorical data by *P*-value and relative risk (RR) and risk difference (RD) with 95% confidence interval; the number needed to treat (NNT) will be calculated if there is statistical significance between groups. All statistical analyses of the data will be performed by using SPSS software version 21.0 (SPSS Inc., Chicago, IL, USA).

## Discussion

Although we have confirmed that the combination of manual and low frequency EA can improve insulin sensitivity, hormone levels, and anxiety situation in patients with PCOS by the prospective study (ClinicalTrials.gov NCT02026323) [25], it had the limitation of being a single center study without comparison groups. There is still a lack of high-quality studies to determine the effect of acupuncture on insulin sensitivity in PCOS women and IR.

Based on the results of the prospective observational study (ClinicalTrials.gov NCT02026323) [25], we designed this randomized, double-blind, placebo-controlled, and multi-center study protocol to evaluate whether acupuncture treatment is more effective than metformin and sham acupuncture in improving the insulin sensitivity of PCOS and IR. The use of a larger sample size in a randomized setting, controlled by sham acupuncture and common medicine of insulin-sensitizing agents, will reduce the risk of type II error. Therefore, the results of this study may provide evidence for using acupuncture in future clinical practice in this area.

### Trial status

The study was registered at ClinicalTrials.gov on 24 June 2015. Recruitment was started on 11 November 2015 and is expected to end in March 2018. Recruitment is ongoing and 114 participants were randomized as of the end of December 2016. The final report will be available in 2018.

## Additional file

Additional file 1: SPIRIT 2013 checklist. Recommended items to address in a clinical trial protocol and related documents. (PDF 106 kb)
